# A commentary on ‘Alteration of the fecal microbiome in patients with cholecystectomy: potential relationship with postcholecystectomy diarrhea – before and after study’

**DOI:** 10.1097/JS9.0000000000000870

**Published:** 2023-11-02

**Authors:** Jiayi Ye, Yanjie Hu, Ka Li

**Affiliations:** West China Hospital, Sichuan University/West China School of Nursing, Sichuan University, Chengdu, People’s Republic of China

*Dear Editor*,

Gallstones (GS) are among the most prevalent benign gastrointestinal disorders globally, and cholecystectomy is the gold standard treatment for symptomatic GS^1^. Although cholecystectomy offers notable advantages in the treatment of gallstone-related diseases, the biochemical and physiological changes due to cholecystectomy can lead to clinical symptoms such as abdominal pain and diarrhea^2^, which can seriously affect the quality of life of patients and impede their recovery process. However, the underlying mechanism remains unclear. We read with interest the study published in the *International Journal of Surgery* by Noh *et al*.^3^. This study explored alterations in the fecal microbiome of cholecystectomy patients and their potential relationship with postcholecystectomy diarrhea (PCD). The results of the study indicated that the fecal microbiome composition of GS patients was different from that of healthy controls (HC), and that cholecystectomy did not significantly affect the fecal microbiome at 3 months postoperatively. Most importantly, this study identified key flora associated with PCD. These findings are both timely and of great significance. This offers the possibility of restoring the fecal microbiome to alleviate PCD. While the rigorous efforts and valuable contributions of this study are deeply appreciated, some constructive suggestions are offered for further refinement.

First, the inclusion–exclusion criteria for studies. The influence of metabolic diseases like diabetes and hyperlipidemia on the fecal microbiome is well-established^4,5^. Given that these diseases alter the composition of the fecal microbiome, it may be prudent to exclude GS patients with these diseases or at least take these diseases into account in the analysis, thereby increasing the robustness of the results of the comparison of the fecal microbiome of GS patients with those of HC, reducing confounding of results by confounding factors.

Second, baseline characteristics of the PCD (−) and PCD (+) groups. Previous studies have found that age, body mass index, and sex may be associated with PCD^6^. It would be beneficial to ensure a balance in these baseline characteristics between the PCD (−) and PCD (+) groups. This would improve the robustness of the study findings in terms of fecal microbiome differences between these two groups.

Third, the time of occurrence of PCD. It would be interesting to know if there is a difference in the fecal microbiome composition based on the timing of PCD occurrence. For instance, do patients who develop PCD immediately after surgery have a different microbiome profile than those who develop it several weeks postoperatively?

In conclusion, we thank the study by Noh *et al*. This study is an important step in our understanding of the relationship between the fecal microbiome and PCD. The intricate association between the fecal microbiome and PCD highlights the importance and necessity of further research in this domain. Such research could pave the way for better prevention and treatment strategies to benefit more people. My suggestions are merely to refine an already outstanding piece of research further. I eagerly look forward to the authors’ follow-up research in this important domain.

## Ethical approval

It does not need ethical approval because this is a letter.

## Consent

It does not need consent because this is a letter.

## Sources of funding

The comment does not receive any funding.

## Author contribution

J.Y. and Y.H.: methodology, formal analysis, and writing – original draft; K.L.: conceptualization, methodology, supervision, and writing – review and editing.

## Conflicts of interest disclosure

There are no conflicts of interest.

## Research registration unique identifying number (UIN)

Our study did not involve human subjects.

## Guarantor

Ka Li.

## Provenance and peer review

Not commissioned, externally peer-reviewed.

## Data availability statement

No data was generated during the writing of this study.
